# Correction: High-energy X-ray phase-contrast CT of an adult human chest phantom

**DOI:** 10.1038/s41598-025-24931-7

**Published:** 2025-10-28

**Authors:** Jannis N. Ahlers, Lorenzo D’Amico, Henriette Bast, Lucy F. Costello, Martin Donnelley, Samantha J. Alloo, Stephanie A. Harker, Ying Ying How, Michelle K. Croughan, James A. Pollock, Daniel Häusermann, Anton Maksimenko, Christopher Hall, Timur E. Gureyev, Yakov I. Nesterets, Marcus J. Kitchen, Konstantin M. Pavlov, Kaye S. Morgan

**Affiliations:** 1 School of Physics and Astronomy, Monash University, 3800 Clayton, VIC Australia; 2 Elettra-Sincrotrone Trieste S.C.p.A., Trieste, Italy; 3 Chair of Biomedical Physics, School of Natural Sciences, Technical University of Munich, 85748 Garching, Germany; 4 Munich Institute of Biomedical Engineering, 85748 Garching, Germany; 5 Adelaide Medical School and Robinson Research Institute, University of Adelaide, Adelaide, South Australia Australia; 6 Respiratory Medicine, Women’s and Children’s Hospital Adelaide, Adelaide, South Australia Australia; 7 Australian Synchrotron, ANSTO, 3168 Clayton, VIC Australia; 8 School of Physics, University of Melbourne, 3010 Parkville, Victoria Australia; 9 Commonwealth Scientific and Industrial Research Organisation, 3168 Clayton, VIC Australia; 10 School of Physical and Chemical Sciences, University of Canterbury, 8140 Christchurch, New Zealand; 11 School of Science and Technology, University of New England, 2351 Armidale, NSW Australia

Correction to: *Scientific Reports* 10.1038/s41598-025-14956-3, published online 11 August 2025

The original version of this Article contained an error in the name of the author Lorenzo D’Amico, which was incorrectly given as Lorenzo D. Amico.

The original Article has been corrected.

