# Supplementary material for: High-energy X-ray phase-contrast CT of an adult human chest phantom
Source: Sci Rep. 2025 Aug 11;15:29314. doi: 10.1038/s41598-025-14956-3 (PMC12340022; doi:10.1038/s41598-025-14956-3)
Supplement: Supplementary file 1 — Supplementary Information. [file 41598_2025_14956_MOESM1_ESM.pdf]

# High-energy X-ray phase-contrast CT of an adult human chest phantom: Supplemental document

## S1 Quality dependence on dose

The imaging quality metrics that were used in the main paper included a dose normalisation in order to allow for the comparison of quantities across different CT slices. In order to check whether this normalisation correctly removed any dependence on dose, we plotted the metrics against dose in Fig. S1. Overall, the plots are flat with no significant dependence evident. The imaging quality analysis was repeated on measurements with a mean absorbed dose to soft tissue below 50 mGy. The results are shown in Fig. S2. As before, the best results are achieved at 70 keV and 7.5 m.

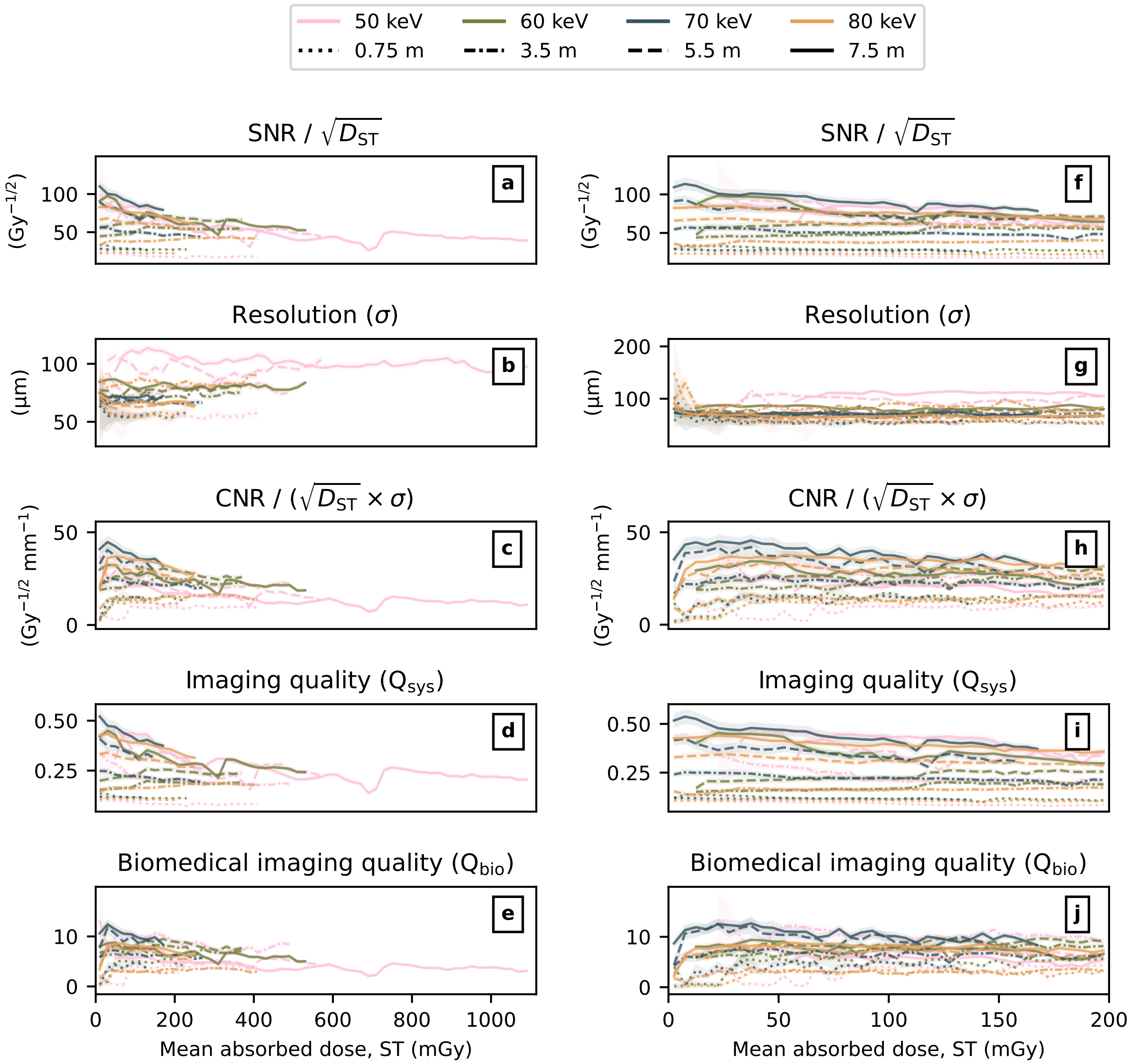


**Fig. S1.** Dose dependence of imaging quality metrics. On the left, the full range of doses is plotted. On the right, the range is restricted to below 200 mGy.

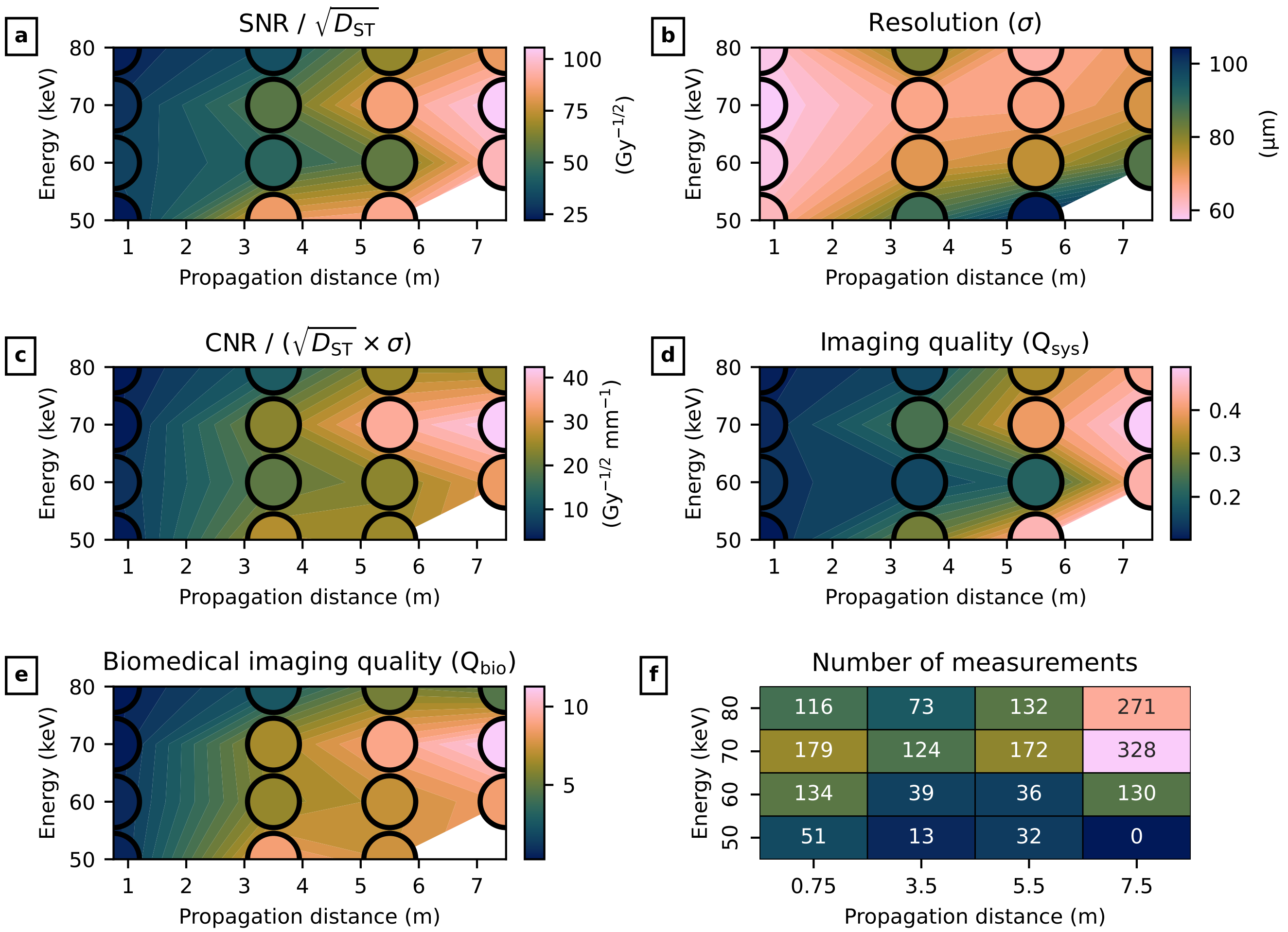


**Fig. S2.** Results of imaging quality analysis at low dose (< 50 mGy). At 50 keV and 7.5 m there were no slices with a mean absorbed dose below 50 mGy, all were at higher dose. In the combined imaging metrics, the peak image quality is still at 70 keV and 7.5 m.
